# Comparative experimental investigation on the efficacy of mono- and multiprobiotic strains in non-alcoholic fatty liver disease prevention

**DOI:** 10.1186/s12876-016-0451-2

**Published:** 2016-03-15

**Authors:** Nazarii Kobyliak, Tetyana Falalyeyeva, Oleksandr Virchenko, Galyna Mykhalchyshyn, Petro Bodnar, Mykola Spivak, Dmytro Yankovsky, Tetyana Beregova, Lyudmyla Ostapchenko

**Affiliations:** Bogomolets National Medical University, T. Shevchenko boulevard, 13, Kyiv, 01601 Ukraine; Taras Shevchenko National University of Kyiv, Volodymyrska Str., 64/13, Kyiv, 01601 Ukraine; Zabolotny Institute of Microbiology and Virology, National Academy of Sciences of Ukraine, Zabolotny Str., 154, Kyiv, 03680 Ukraine; Scientific and Production Company “O.D. Prolisok”, Kyiv, Ukraine

**Keywords:** NAFLD, Prevention, Obesity, Lyophilized and alive probiotic strains, Monoprobiotic, Multistrain probiotics

## Abstract

**Background:**

To investigate the efficacy of different probiotic strains, their combinations and forms (alive or lyophilized) in nonalcoholic fatty liver disease (NAFLD) prevention.

**Methods:**

In this study, 70 rats have been used divided into 7 groups of 10 animals in each: I – intact rats, II-VII – rats with monosodium glutamate (MSG)-induced NAFLD. Rats with NAFLD were untreated (group II, MSG-obesity group) and treated with probiotics (groups III–VII). In order to develop NAFLD, newborn rats of groups II–VII were injected with a solution of monosodium glutamate (MSG) (4 mg/g) subcutaneously (s.c.) at 2nd,4th, 6th, 8th,10th postnatal day. The groups III–V received lyophilized monoprobiotics *B. animalis VKL*, *B. animalis VKB*, *L.casei IMVB-7280,* respectively. The group VI received 2.5 ml/kg of an aqueous solution of a mixture of the three probiotic strains (2:1:1 *Lactobacillus casei IMVB-7280, Bifidobacterium animalis VKL, Bifidobacterium animalis VKB*) at a dose of 50 mg/kg (5 × 10^9^ CFU/kg) (g) (intragastrically). The group VII was treated with multiprobiotic “Symbiter” containing biomass of 14 alive probiotic strains (*Lactobacillus* + *Lactococcus* (6 × 10^10^ CFU/g), *Bifidobacterium* (1 × 10^10^/g), *Propionibacterium* (3 × 10^10^/g), *Acetobacter* (1 × 10^6^/g)) at a dose of 140 mg/kg (1.4 × 10^10^ CFU/kg). The treatment with probiotics was started at the age of 1 month. There were 3 courses of treatment, each included 2-week administration and 2-week break. All parameters were measured in 4-month aged rats.

**Results:**

Introduction of MSG during the neonatal period leads to the NAFLD development in the 4-months old rats. For steatosis degree there was no significant difference between MSG-obesity group and lyophilized monocomponent probiotics groups (III–V). The highest manifestation of steatosis was observed for *B. animalis VKL* group (2.0 ± 0.25) as compared to *B. animalis VKB* (1.70 ± 0.21) and *L. casei IMVB-7280* (1.80 ± 0.20). The steatosis score changes between all monoprobiotics groups (III–V) were insignificant. Administration from birth of both alive (VII) and lyophilized (VI) probiotic mixture lead to a significant decrease by 69.5 % (*p* < 0.001) and 43.5 % (*p* < 0.025) of steatosis score respectively as compared to the MSG-obesity group (2.3 ± 0.21 %). For both alive and lyophilized probiotic mixtures, reduction of lobular inflammation was observed. These histological data were confirmed by the significant decrease of total lipids and triglycerides content in the liver approximately by 22–25 % in groups treated with probiotic mixtures (VI, VII) compared to the MSG-obesity group.

**Conclusion:**

We established failure of NAFLD prevention with lyophilized monoprobiotic strains and the efficacy of probiotic mixture with the preference of alive probiotic strains.

## Background

Non-alcoholic fatty liver disease (NAFLD) ranges from simple steatosis to non-alcoholic steatohepatitis (NASH) that can have different degrees of fibrosis and progress to liver cirrhosis and hepatocellular carcinoma [1]. NAFLD that is associated with obesity and type 2 diabetes has a prevalence of 15–20 % in the general population and 76–90 % in the obese [2]. NAFLD is currently a leading cause of chronic liver disease [3, 4], which has resulted in significant health concerns such as morbidity, mortality, and liver transplants [5].

According to Day’s two-hit model of NAFLD pathogenesis insulin resistance as the first hit, causes lipid accumulation in hepatocytes and leads to the development of fatty liver. The second hit includes cellular insults such as oxidative stress and lipid oxidation, which damages the liver cells and triggers an inflammatory process that leads to pathological changes in hepatocytes that result in NASH [6]. Recent studies present a clear evidence that gut microbiota are strongly implicated in NAFLD pathogenesis and progression through several mechanisms [7].

The underlying mechanisms, which link altered gut microbiota composition to NAFLD are modulation of dietary choline metabolism [8] and production of endogenous ethanol [9], increased gut permeability [10] with subsequent endotoxemia and metabolic low-grade inflammation [11], increased energy harvest from the diet [12] and impaired short-chain fatty acids synthesis [13], decreased absorbtion of vitamins and biologically active compounds [12], altered bile acids metabolism, and FXR/TGR5 signaling [14]. Prebiotics and probiotics have physiologic functions that contribute to the health of gut microbiota and/or restoration of microflora, maintenance of a healthy body weight and control of factors associated with NAFLD through the various above mentioned pathways [15].

In our previous work, we have shown that periodic treatment with multiprobiotic containing biomass of 14 alive strains (*Lactobacillus, Lactococcus*, *Bifidobacterium*, *Propionibacterium*, *Acetobacter*) prevents, at least partially, the MSG-induced obesity [16] and NAFLD development [17]. However the question regarding the efficacy of different probiotic strains, their combination and form (alive or lyophilized) in the management of NAFLD remains open, which formed the current study aims.

## Methods

### Animals

This study was carried out in strict accordance with the recommendations in the Guide for the Care and Use of Laboratory Animals of the National Institutes of Health and the general ethical principles of animal experiments, approved by the First National Congress on Bioethics Ukraine (September 2001). The protocol was approved by the Committee on the Ethics of Animal Experiments of the Taras Shevchenko National University of Kyiv (Protocol number: 10/2014). The rats were kept in collective cages under controlled conditions of temperature (22 ± 3 °C), light (12 h light/dark cycle) and relative humidity (60 ± 5 %). The animals were fed laboratory chow (PurinaW) and tap water ad libitum.

### Experiment design

**Fig. 1:**
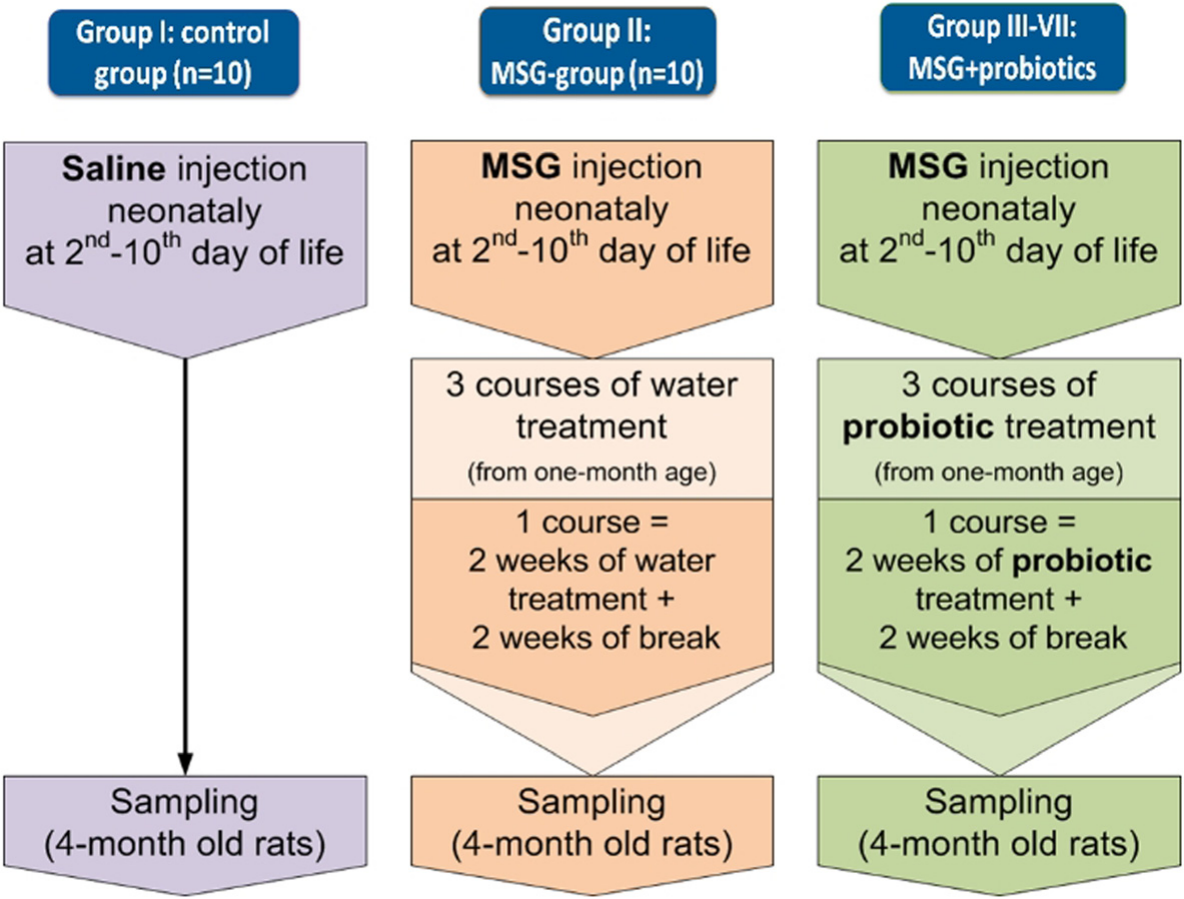
Experiment design

The study included 70 male Wistar rats divided into 7 groups, of 10 animals in each (Fig. 1). Newborns rats of the control group (I) were administered with saline subcutaneously (s.c.) in the volume of 8 μl/g at 2nd, 4th, 6th, 8th and 10th postnatal days. Newborns rats of groups II–VII were injected with monosodium glutamate solution (MSG) (4.0 mg/g of body weight) s.c. at 2nd, 4th, 6th, 8th and 10th postnatal days [18]. Neonatal administration of MSG causes the significant accumulation of fat in the abdomen of the adult rats. This happens because of the neurotoxicity effects on the arcuate and ventromedial nuclei of the hypothalamus [19]. In our previous work, we have shown the development of NAFLD under conditions of the severe visceral obesity induced by MSG [17]. Thus, the obtained results confirmed the validity of the usage of MSG for NAFLD development.

The groups III–VII were treated with probiotics. The groups III–V received lyophilized monoprobiotics *B. animalis VKL*, *B. animalis VKB*, *L. casei IMVB-7280* respectively. The group VI received the mix of these three probiotic strains. The group VII was treated with multiprobiotic “Symbiter” which was supplied by Scientific and Production Company “O.D. Prolisok”. It contains of 14 alive probiotic strains of *Lactobacillus* + *Lactococcus (6 × 10*^*10*^ CFU/g*)*, *Bifidobacterium (1 × 10*^*10*^/g*)*, *Propionibacterium (3 × 10*^*10*^/g*)*, *Acetobacter (1 × 10*^*6*^/g*)* genera.

Administration was started at the end of the 4th week after birth and continued intermittently by alternating a 2-week course and 2 weeks intervals of nontreatment. Within 4 months after birth rats were on a normal diet. All parameters were measured in 4-months old rats.

### Sample collection and blood biochemistry analysis

Rats of all groups were fasted for approximately 12 h prior sacrifice. Rats were sacrificed by cervical dislocation under urethane anesthesia. Blood was drawn from the apex of the cardiac ventricle and few blood drops were collected into a microcentrifuge tube containing a mixture of NaF and EDTA at a 2:1 (*w/w*) ratio. Blood sample was collected into a sterile tube and centrifuged at 3500 rpm (2260 g) for 15 min. After centrifugation serum supernatant for further analysis was aliquoted into microcentrifuge tubes and stored at −80 °C. Bilirubin, activity of alanine and aspartate aminotransferase in serum were determined by the standard biochemical methods.

### Liver histology assessment

For histological analysis liver tissue samples from both the right and left hepatic lobes were taken (sample size 0.5 × 0.5 cm). After fixation for 24 h in a liquid Buena, liver fragments were dehydrated in alcohol of increasing concentrations (from 70 to 96 °), embedded in paraffin and then cut with a thickness of 5–6 microns and stained with hematoxylin-eosin. A pathologist blinded to group distributions performed the histological analyses of slides using light microscopy («Olympus», Japan). To assess morphological changes in the liver we used NAS (NAFLD activity score), which includes histological features and has been defined as unweighted sum of scores for steatosis (0–3), lobular inflammation (0–3) and ballooning (0–2). Acording to NAS scores ≥5 are diagnosed as non-alcoholic steatohepatitis (NASH), and cases with a NAS <3 are mentioned as not NASH [20]. Lipid extraction from the liver was performed according to Folch et al. [21].

### Statistical analysis

Statistical analysis was performed with the SPSS-20 software. All data in this study were expressed as means ± standard error of the mean (M ± SEM) or %. Data distribution was analyzed using the Kolmogorov-Smirnov normality test. Continuous variables with parametric distribution were analyzed using Analysis of Variance (ANOVA) and if the results were significant, a post-hoc Tukeys test was performed. For data with non-parametric distribution Kruskall-Wallis and post-hoc Dunn’s test were conducted for multiple comparisons. For comparisons of categorical variables we conducted *χ*^2^ test. The difference between groups was defined to be statistically significant when a *p*-value was less than 0.05.

## Results

We didn’t find any significant difference in biochemical indicators (alanine transaminase (*ALT*), aspartate transaminase (*AST*), bilirubin) of liver in blood serum between intact, MSG-obesity and MSG-probiotics group (Table 1).

**Table 1:** Liver function tests of rats under MSG-induced obesity and probiotic correction

Parameters	Control rats (*n* = 10)	MSG-induced obesity (*n* = 10)	*B. animalis* VKL (*n* = 10)	*B. animalis* VKB (*n* = 10)	*L. casei* IMVB-7280 (*n* = 10)	Poliprobiotics (*n* = 10)	Symbiter (*n* = 10)
Total bilirubin, umol/l	12.9 ± 0.73	12.6 ± 0.58	13.6 ± 0.68	12.5 ± 0.3	13.3 ± 0.63	12.4 ± 0.47	13.4 ± 0.63
Indirect bilirubin, umol/l	8.3 ± 0.55	8.2 ± 0.35	8.6 ± 0.47	8.2 ± 0.29	8.5 ± 0.5	8.3 ± 0.33	8.3 ± 0.53
Direct bilirubin, mmol/l	4.6 ± 0.33	4.4 ± 0.3	5.0 ± 0.29	4.3 ± 0.26	4.8 ± 0.29	4.1 ± 0.27	5.1 ± 0.23
ALT, mkkat/l	0.231 ± 0.011	0.243 ± 0.015	0.230 ± 0.013	0.209 ± 0.010	0.221 ± 0.008	0.211 ± 0.014	0.212 ± 0.007
AST, mkkat/l	0.386 ± 0.007	0.397 ± 0.011	0.396 ± 0.015	0.382 ± 0.011	0.389 ± 0.014	0.373 ± 0.016	0.381 ± 0.018

Data are presented as the M ± SEM. One-way ANOVA or Kruskall-Wallis test were performed for data analysis. All differences were not significant (*p* > 0.05)

**Fig. 2:**
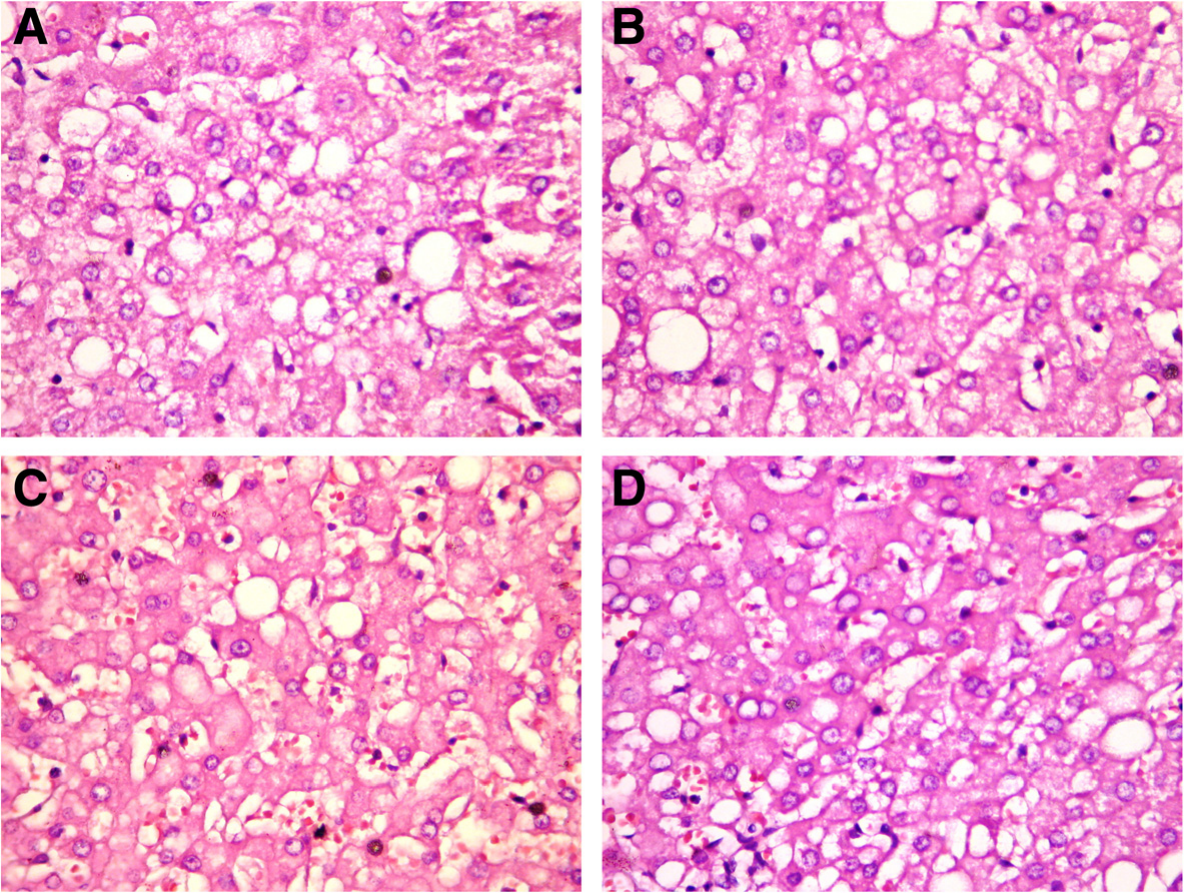
Light microscopic micrographs of the rat liver tissue stained with hematoxylin and eosin, ×400. In micrographs predominantly microvesicular pronounced total steatosis were mainly observed. **a** - MSG-induced obesity group; **b** - B. *animalis* VKL group; **c** - B. *animalis* VKB group; **d** - *L. casei* IMVB-7280 group

**Fig. 3:**
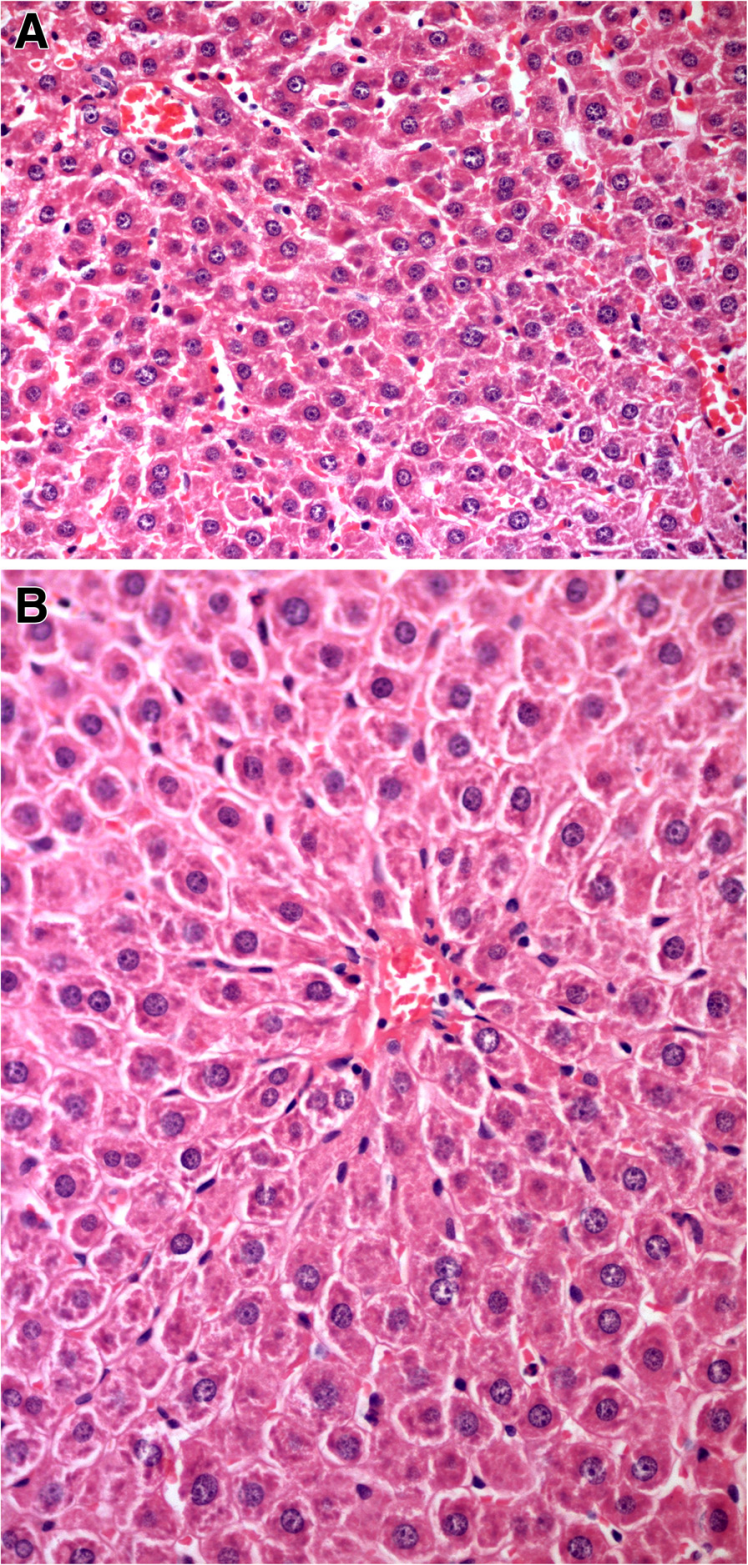
Light microscopic micrographs of the rat liver tissue stained with hematoxylin and eosin, ×400. In micrographs focal mild microvesicular steatosis were mainly observed. **a** - Poliprobiotics group; **b** - Symbiter group

Liver histology changes, as assessed by the NAS score, associated with the administration of different types of probiotics are represented in Table 2. For steatosis degree there was no significant difference between the MSG-obesity group and lyophilized monocomponent probiotics groups (III–V) (Fig. 2a-d). The highest manifestation of steatosis was observed for *B. animalis VKL* group (2.0 ± 0.25) as compared to *B. animalis VKB* (1.70 ± 0.21) and *L. casei IMVB-7280* (1.80 ± 0.20). The steatosis score changes between all monoprobiotics groups (III–V) were insignificant. Administration from birth of both alive (VII) and lyophilized (VI) probiotic mixture lead to a significant decrease by 69.5 % (*p* < 0.001) and 43.5 % (*p* = 0.025) of steatosis score respectively as compared to the MSG-obesity group (2.3 ± 0.21) (Fig. 3a and b).

**Table 2:** Morphological changes in the rat liver assessed by NAFLD activity score (NAS)

Parameters	Intact rats (*n* = 10)	MSG-induced obesity (*n* = 10)	B. *animalis* VKL (*n* = 10)	B. *animalis* VKB (*n* = 10)	*L. casei* IMVB-7280 (*n* = 10)	Poliprobiotics (*n* = 10)	Symbiter (*n* = 10)
Steatosis (0–3)	0.10 ± 0.1^a^	2.30 ± 0.21^d^	2.0 ± 0.25^cd^	1.70 ± 0.21^cd^	1.80 ± 0.20^cd^	1.30 ± 0.3^bc^	0.70 ± 0.15^ab^
Lobular inflammation (0–2)	0.0 ± 0.0^a^	1.0 ± 0.21^b^	0.60 ± 0.16^ab^	0.40 ± 0.16^ab^	0.40 ± 0.16^ab^	0.30 ± 0.15^a^	0.10 ± 0.1^a^
Ballooning degeneration (0–2)	0.0 ± 0.0^a^	0.20 ± 0.13^a^	0.10 ± 0.1^a^	0.10 ± 0.1^a^	0.10 ± 0.1^a^	0.0 ± 0.0^a^	0.0 ± 0.0^a^
Total NAS (0–8)	0.0 ± 0.0^a^	3.60 ± 0.4^d^	2.70 ± 0.36^cd^	2.2 ± 0.25^c^	2.30 ± 0.21^c^	1.60 ± 0.3^bc^	0.80 ± 0.2^ab^
Prevalence of NASH, %	–	30	10	–	–	–	–

Data are presented as the M ± SEM. One-way ANOVA with post hoc Tukeys test for multiple comparisons were performed for data analysis. ^a, b, c, d^ Values at the same row with different superscript letters show significant differences at *p* < 0.05

**Fig. 4:**
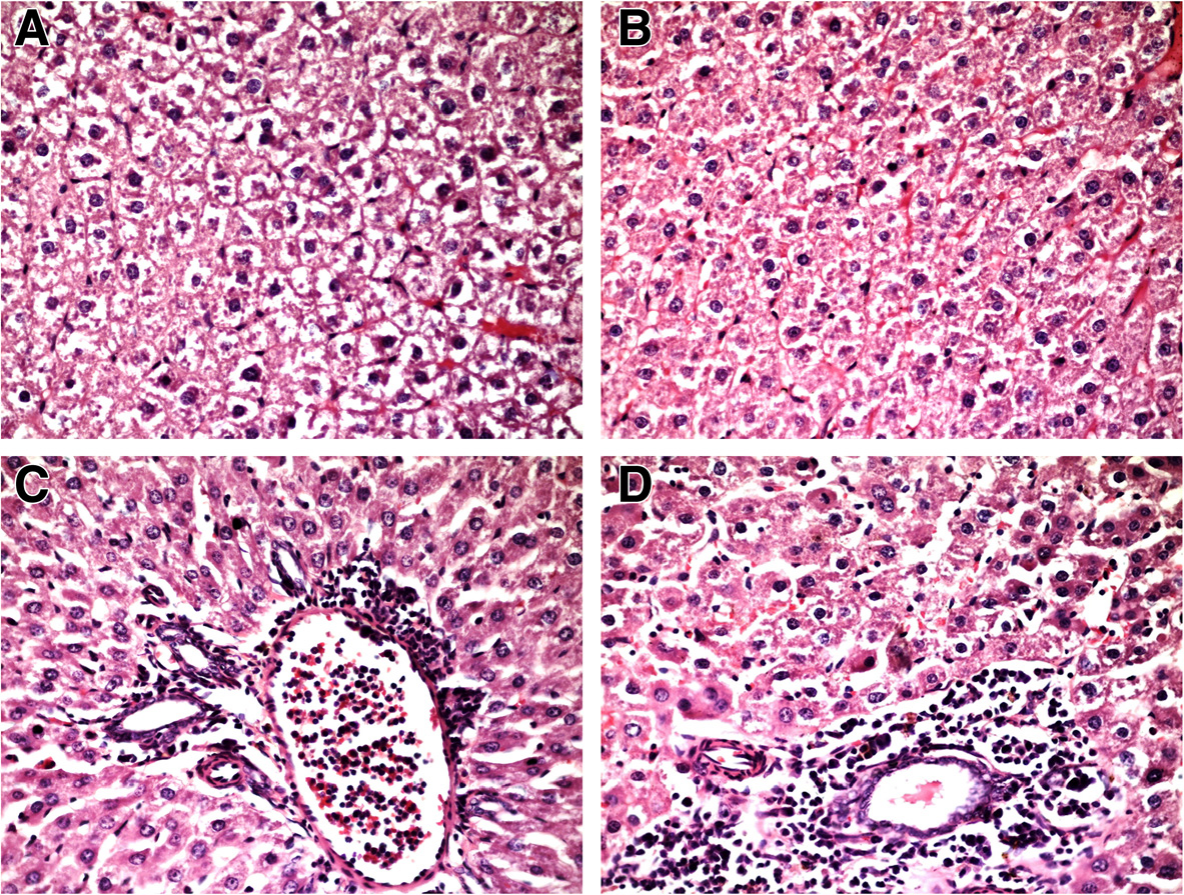
Light microscopic micrographs of the rat liver tissue stained with hematoxylin and eosin, ×400. In micrographs microvesicular steatosis with perivascular leukocyte infiltration at zone 3 (mild lobular inflammation) (**c**, **d**) and focal necrosis as a result of hepatocytes ballooning degeneration – lack of nuclei (in center) (**a**, **b**) was observed. **a** - MSG-induced obesity group; **b** - B. *animalis* VKL group; **c** - B. *animalis* VKB group; **d** - *L. casei* IMVB-7280 group

Similar to steatosis score changes we found that administration of both alive and lyophilized probiotic mixtures from 4th week after birth lead to a significant reduction of liver inflammation manifestation in the adulthood period in rats when compared with littermates with MSG-induced obesity. In the liver histology sections NASH specific lesions were observed. Specifically, the inflammation was mild and predominantly lobular rather than portal, with typically mixed infiltrates, which included chronic inflammatory cell phenotypes, such as lymphocytes, monocytes (Fig. 4a-d). Any significant changes in lobular inflammation as assessed by NAS score between MSG-induced obesity group and lyophilized monocomponent probiotics groups (III–V) were not found. The most prominent changes from all lyophilized groups were described for *B. animalis VKL.* Only in this group we diagnosed NASH in 10 % of animals that was significant (*p* = 0.026) as compared to MSG-obesity group with 30 % of animals with NASH. All groups were typically relative in histological presentation of ballooning degeneration due to the absence of any significant difference (*p* > 0.05) (Table 2).

The lowest total NAS score was observed after administration of alive multiprobiotic group (0.8 ± 0.2) that were insignificant in control rats and statistically lower as compared to all monoprobiotics groups (III–V). When used lyophilized (VI) probiotic mixture changes in total NAS score between other treated were insignificant.

**Fig. 5:**
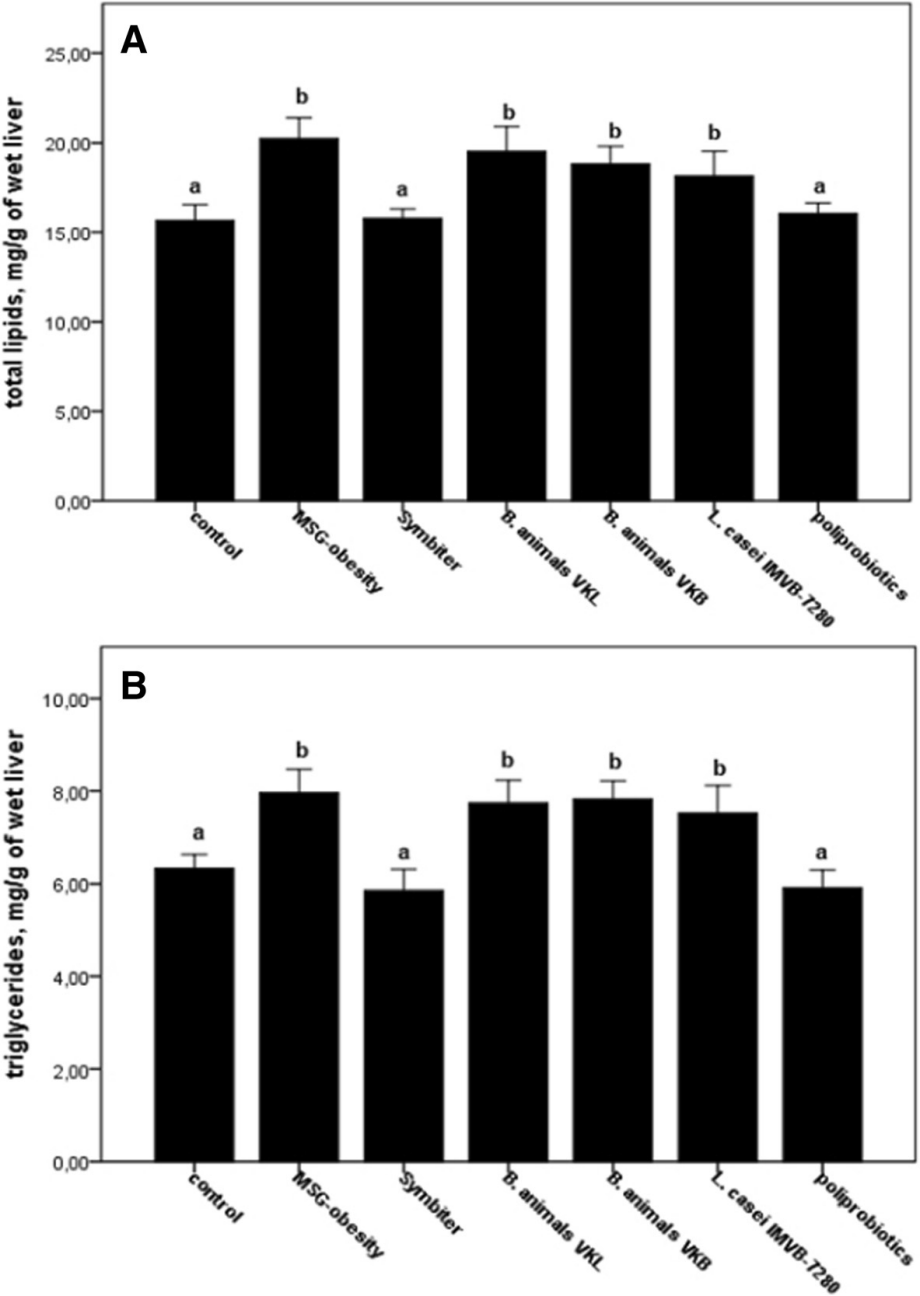
Liver total lipids (**a**) and triglycerides (**b**) content of rats with the MSG-induced obesity and probiotic correction. Data are presented as the M ± SEM. One-way ANOVA with post hoc Tukeys test for multiple comparisons were performed for data analysis. ^a, b^ Values at the same row with different superscript letters show significant differences at *p* < 0.05

In parallel with improving of total NAS score for both policomponent probiotic mixture, we observed a significant decrease of total lipids and triglycerides content in liver approximately by 22–25 % respectively as compared to the MSG-obesity group (Fig. 5a and b). After administration of lyophilized monocomponents probiotics (groups III–V) the changes in the amount of liver lipids were insignificant.

## Discussion

Our study conclusively showed that short-term courses of probiotic mixtures from birth have a preventive effect on fatty liver disease development under condition of the MSG-induced obesity. Nevertheless therapeutic potential are more pronounced for alive probiotic multistrain mixture (VII) because only for Symbiter group significantly lower degree of steatosis and total NAS score were detected as compared to monocomponent probiotics groups (III–V) and any significant changes in liver histology assessment parameters were not found compared with intact rats.

In particular, different *Lactobacillus* та *Bifidobacterium* strains have specific effects on the markers of obesity in rodent models. The analysis of from the literature data published from January 2013 to July 2014 by Cani et al. showed that at least 15 different strains of *Lactobacillus* and 3 strains of *Bifidobacterium* do not equally influence hepatic lipids and NAFLD development in different animal models. Remarkably, 12 strains decreased hepatic tissue inflammation and 11 reduced the hepatic triglyceride content when given as a single treatment [22].

In contrast to our study, where we did not notice any significant single-strain specific changes in lipid metabolism and NAFLD development, a recent study compared the effects of four *Bifidobacteria* strains (*Bifidobacteria* L66-5, L75-4, M13-4 and FS31-12) on lipid metabolism in an high-fat diet obese mice. All the four strains could reduce serum and liver triglyceride and significantly alleviate the lipid deposition in the liver. As for total cholesterol only *Bifidobacteria* L66-5 and *Bifidobacteria* FS31-12 significantly decreased its amount in the liver [23].

Administration of single-strain probiotic of *Lactobacillus rhamnosus GG* protects mice from NAFLD development induced by a high-fructose diet through an increase of beneficial bacteria, restoration of the gut barrier function and subsequent attenuation of liver inflammation and steatosis [24]. Another study demonstrated that treatment with *Lactobacillus rhamnosus GG* for 13 weeks under condition of high fat diet improved insulin sensitivity and reduced lipid accumulation by stimulating adiponectin secretion and consequent activation of AMPK [25].

In addition, it was reported that an oral supplementation of *Bifidobacterium adolescentis* (5 × 10(7) CFU/ml) ad libitum for 12 weeks protected against a diet-induced NASH in C57BL/6 mice. Furthermore, mice treated with probiotic had significantly decreased liver damage, which was associated with prevention from lipid peroxidation, NFκB activation and finally inflammation in the liver [26].

On the other hand, our data find support in another recent study that established that single-strain probiotics of *Lactobacillus curvatus HY7601* significantly reduced liver fat accumulation as compared to *Lactobacillus plantarum KY1032* in diet-induced obesity*.* Combination of this probiotic was more effective for inhibiting gene expressions of various enzymes responsible for fatty acid synthesis in the liver, concomitant with decreases in fatty acid oxidation-related enzyme activities and their gene expressions [27].

Plaza-Diaz in Zucker rats with genetic determined obesity evaluated the effects of *Lactobacillus paracasei CNCM I-4034*, *Bifidobacterium breve CNCM I-4035* and *Lactobacillus rhamnosus CNCM I-4036* probiotic strains and their mixture on the hepatic steatosis development as compared to placebo. In this study only single-strain probiotic *of Lactobacillus rhamnosus or Bifidobacterium breve* and the mixture of *Bifidobacterium breve* and *Lactobacillus paracasei* decreased triacylglycerol content in the rat liver and reduced manifestation of hepatic steatosis in part by lowering serum LPS [28].

The improvement of NAFLD by probiotics strains can be achieved by various pathways. Mei et al. (2015) have shown that decrease in triglycerides level, total cholesterol, free fatty acids, and cholesterol low-density lipoproteins is associated with an increased uptake of cholesterol from the blood, its excretion with bile by the liver, and reduction of cholesterol synthesis under conditions of probiotic treatment of NAFLD [29]. Contrary to our findings, the scientists established the effectiveness of monostrains of *Lactobacillus* species. The influence of probiotics on lipid metabolism was associated with up- and downregulation of certain genes. The team has found an overexpression of low-density lipoproteins receptor that enhances the absorption of low-density lipoproteins by the liver. Besides, cholesterol 7α-hydroxylase activity was elevated that proves the enhanced excretion of cholesterol with bile. Moreover, the farnesoid X receptor was up-regulated indicating the increased bile acid production. The reduction of cholesterol synthesis by probiotics was confirmed by the downregulation of 3-hydroxy-3-methyl glutaryl coenzyme A reductase and sterol regulatory element binding protein-1c. In addition, PPAR-α expression was high and PPAR-γ was low in rats with NAFLD and treated with probiotic strains that diverts lipid metabolism from fat deposition to β-oxidation of fatty acids [29].

Some light on the underlying mechanisms of probiotic impact was shed. It is known that symbiotic bacteria can produce short chain fatty acids, e.g. butyrate. This substance inhibits the activation of toll-like receptor (TLR)-dependent signalling cascades in the liver through strengthening gut tight junction, and reduction of bacterial endotoxin translocation to the liver that was shown on the model of western-style diet-induced NAFLD [30]. It was established that butyrate prevented lipid peroxidation through the reduction of 4-hydroxynonenal protein adducts level and downregulates inducible nitric oxide synthase that is critical for regulation not only NF-κB-depending signalling cascades in the development of NAFLD but also for expression of the TLR-4 adaptor protein myeloid differentiation primary response gene 88. Thus, production of butyrate results in attenuation of inflammation and TLR-dependent signalling in the liver under conditions of experimental NAFLD [30].

## Conclusions

Postnatal administration of both alive (VII) and lyophilized (VI) probiotic mixture lead to significant decrease of hepatic steatosis, total lipids and triglycerides content in the liver as compared to MSG-obesity and may be more beneficial than single-strain probiotics. Thus, multicomponent probiotics have a preventive effect on fatty liver disease development. It may be related to more pronounced viability of the alive strains and their prevention of bacterial translocation. Multistrain or multispecies formed mutualistic interactions in mixtures and therefore were able to share with different metabolites, affect different receptors and produced various biologically active compounds. So, their synergistic overall effect is greater than the sum of their individual effects. On the other hand, most likely due to different putative mechanisms of action, strain-specific probiotics must be considered for novel investigation in different metabolic diseases.

## Abbreviations

ANOVA: Analysis of Variance
NAFLD: non-alcoholic fatty liver disease
NASH: non-alcoholic steatohepatitis
TLR: toll-like receptor

## References

[1] Kobyliak N, Abenavoli L (2014). The role of liver biopsy to assess non-alcoholic fatty liver disease. Rev Recent Clin Trials.

[2] Angulo P (2002). Medical progress: nonalcoholic fatty liver disease. N Engl J Med.

[3] Farrell GC, Larter CZ (2006). Nonalcoholic fatty liver disease: from steatosis to cirrhosis. Hepatology.

[4] Weston SR, Leyden W, Murphy R, Bass NM, Bell BP, Manos MM (2005). Racial and ethnic distribution of nonalcoholic fatty liver in persons with newly diagnosed chronic liver disease. Hepatology.

[5] Musso G, Gambino R, Cassader M, Pagano G (2011). Meta-analysis: natural history of nonalcoholic fatty liver disease (NAFLD) and diagnostic accuracy of non-invasive tests for liver disease severity. Ann Med.

[6] Day CP, James OF (1998). Steatohepatitis: a tale of two “hits”?. Gastroenterology.

[7] Arslan N (2014). Obesity, fatty liver disease and intestinal microbiota. World J Gastroenterol.

[8] Buchman AL, Dubin MD, Moukarzel AA, Jenden DJ, Roch M, Rice M (1995). Choline deficiency: a cause of hepatic steatosis during parenteral nutrition that can be reversed with intravenous choline supplementation. Hepatology.

[9] Volynets V, Küper MA, Strahl S, Maier IB, Spruss A, Wagnerberger S (2012). Nutrition, intestinal permeability, and blood ethanol levels are altered in patients with nonalcoholic fatty liver disease (NAFLD). Dig Dis Sci.

[10] Kashyap PC, Marcobal A, Ursell LK, Smits SA, Sonnenburg ED, Costello EK (2013). Genetically dictated change in host mucus carbohydrate landscape exerts a diet-dependent effect on the gut microbiota. Proc Natl Acad Sci U S A.

[11] Cani PD, Bibiloni R, Knauf C, Waget A, Neyrinck AM, Delzenne NM (2008). Changes in gut microbiota control metabolic endotoxemia-induced inflammation in high-fat diet-induced obesity and diabetes in mice. Diabetes.

[12] Turnbaugh PJ, Ley RE, Mahowald MA, Magrini V, Mardis ER, Gordon JI (2006). An obesity-associated gut microbiome with increased capacity for energy harvest. Nature.

[13] Le Poul E, Loison C, Struyf S, Springael JY, Lannoy V, Decobecq ME (2003). Functional characterization of human receptors for short chain fatty acids and their role in polymorphonuclear cell activation. J Biol Chem.

[14] Jiang C, Xie C, Li F, Zhang L, Nichols RG, Krausz KW (2014). Intestinal farnesoid X receptor signaling promotes nonalcoholic fatty liver disease. J Clin Invest.

[15] Kobyliak N, Conte C, Cammarota G, Haley AP, Styriak I (2016). Probiotics in prevention and treatment of obesity: a critical view. Nutr Metab.

[16] Savcheniuk O, Kobyliak N, Kondro M, Virchenko O, Falalyeyeva T, Beregova T (2014). Short-term periodic consumption of multiprobiotic from childhood improves insulin sensitivity, prevents development of non-alcoholic fatty liver disease and adiposity in adult rats with glutamate-induced obesity. BMC Complement Altern Med.

[17] Kondro M, Kobyliak N, Virchenko O, Falalyeyeva T, Beregova T, Bodnar P (2014). Multiprobiotic therapy from childhood prevents the development of nonalcoholic fatty liver disease in adult monosodium glutamate-induced obese rats. Curr Issues Pharm Med Sci.

[18] Kondro M, Mykhalchyshyn G, Bodnar P, Kobyliak N, Falalyeyeva T (2013). Metabolic profile and morpho-functional state of the liver in rats with glutamate-induced obesity. Curr Issues Pharm Med Sci.

[19] Nakagawa T, Ukai K, Ohyama T, Gomita Y, Okamura H (2000). Effects of chronic administration of sibutramine on body weight, food intake and motor activity in neonatally monosodium glutamate-treated obese female rats: relationship of antiobesity effect with monoamines. Exp Anim.

[20] Kleiner DE, Brunt EM, Van Natta M, Behling C, Contos MJ, Cummings OW (2005). Design and validation of a histological scoring system for nonalcoholic fatty liver disease. Hepatology.

[21] Folch J, Lees M, Stanley GHS (1957). A simple method for the isolation and purification of total lipids from animal tissues. J Biol Chem.

[22] Cani PD, Van Hul M (2014). Novel opportunities for next-generation probiotics targeting metabolic syndrome. Curr Opin Biotechnol.

[23] Yin YN, Yu QF, Fu N, Liu XW, Lu FG (2010). Effects of four Bifidobacteria on obesity in high-fat diet induced rats. World J Gastroenterol.

[24] Ritze Y, Bárdos G, Claus A, Ehrmann V, Bergheim I, Schwiertz A (2014). Lactobacillus rhamnosus GG protects against non-alcoholic fatty liver disease in mice. PLoS One.

[25] Kim SW, Park KY, Kim B, Kim E, Hyun CK (2013). Lactobacillus rhamnosus GG improves insulin sensitivity and reduces adiposity in high-fat diet-fed mice through enhancement of adiponectin production. Biochem Biophys Res Commun.

[26] Reichold A, Brenner SA, Spruss A, Förster-Fromme K, Bergheim I, Bischoff SC (2014). Bifidobacterium adolescentis protects from the development of nonalcoholic steatohepatitis in a mouse model. J Nutr Biochem.

[27] Yoo SR, Kim YJ, Park DY, Jung UJ, Jeon SM, Ahn YT (2013). Probiotics L. plantarum and L. curvatus in combination alter hepatic lipid metabolism and suppress diet-induced obesity. Obesity (Silver Spring).

[28] Plaza-Diaz J, Gomez-Llorente C, Abadia-Molina F, Saez-Lara MJ, Campaña-Martin L, Muñoz-Quezada S (2014). Effects of Lactobacillus paracasei CNCM I-4034, Bifidobacterium breve CNCM I-4035 and Lactobacillus rhamnosus CNCM I-4036 on hepatic steatosis in Zucker rats. PLoS One.

[29] Mei L, Tang Y, Li M, Yang P, Liu Z, Yuan J, Zheng P (2015). Co-administration of cholesterol-lowering probiotics and anthraquinone from Cassia obtusifolia L. ameliorate non-alcoholic fatty liver. PLoS One.

[30] Jin C, Sellmann C, Engstler AJ, Ziegenhardt D, Bergheim I (2015). Supplementation of sodium butyrate protects mice from the development of non-alcoholic steatohepatitis (NASH). Br J Nutr.

